# A Comparative Study of the Modal Response of Additively and Subtractively Manufactured Thin Plates After Thermal Loading

**DOI:** 10.1007/s11340-024-01130-5

**Published:** 2025-01-23

**Authors:** M. Weihrauch, P. Lambert, J. Lambros, C. J. Sutcliffe, E. A. Patterson

**Affiliations:** 1 Department of Mechanical and Aerospace Engineering, University of Liverpool, Liverpool, UK; 2 Department of Aerospace Engineering, University of Illinois Urbana-Champaign, Urbana, USA; 3 Meta Consulting LDA, Caldas da Rainha, Caldas da Rainha, Portugal

**Keywords:** Modal Analysis, Thermal Loading, Thin Plates, Additive Manufacturing

## Abstract

**Background:**

Additively-manufactured parts contain residual stresses induced by manufacturing. These residual stresses can be relaxed or redistributed by thermal loading. The presence of internal stress influences the dynamic response of parts, and this is of particular interest in thin plates subject to thermoacoustic loading in hypersonic vehicles and fusion reactors.

**Objective:**

To measure the changes in shape and modal frequencies caused by thermal loading of geometrically-reinforced thin plates that were additively manufactured in Inconel 625.

**Methods:**

Plates were additively-manufactured in landscape and portrait orientations using laser powder bed fusion. The plates were heated to a nominal temperature of 820 °C, which was expected to alleviate the residual stress from the build process. Pre- and post-heating, their modal frequencies were found experimentally and pulsed-laser stereo (3D) digital image correlation was used to evaluate their modal shapes. The resultant modal frequencies and shapes were compared with those from a subtractively-manufactured plate.

**Results:**

It was found that the heat cycle changed the shape of the plates relative to their as-manufactured state in addition to changing their natural frequencies and modal shapes.

**Conclusions:**

The change in shape induced by heating caused shifts in the natural frequencies and changes in the corresponding modal shapes. The results show quantitatively for the first time the important role that residual stresses can play in the dynamic response of geometrically-reinforced thin plates manufactured by additive and subtractive processes.

**Supplementary Information:**

The online version contains supplementary material available at 10.1007/s11340-024-01130-5.

## Introduction

 Additive manufacturing (AM) is an alternative to more traditional subtractive manufacturing methods, enabling the production of parts with complex geometries and reduced waste material. In the aerospace industry laser powder bed fusion (L-PDF), has been identified as an AM technique with great potential due to its ability to produce dense parts with complex geometries and fine features [1]. However, as parts are built layer by layer, they can exhibit anisotropic behaviour and contain large amounts of residual stress, which can lead to warping, especially of thin parts, and defects [1]. To alleviate some of these concerns, components usually undergo a heat treatment after being built to relieve stresses and allow grain regrowth [2].

Understanding the mechanical and dynamic response of AM components as compared to subtractively manufactured ones, especially in demanding environments, is of importance. For instance, in hypersonic flight components are exposed to high aeroacoustic loads in addition to high temperatures originating from the engine and aerothermal loads [3]. Similarly, in fusion energy reactors, plasma facing components, such as divertors, are also exposed to thermomechanical loading [4]. In this study, the effect of thermal loading on the modal response of additively manufactured thin plates was investigated. The plates were geometrically-reinforced to mimic aircraft skin which is reinforced by ribs and stringers. Changes in the modal response of additively manufactured specimens as a result of heating were observed and compared to a subtractively manufactured counterpart.

The anisotropy present in AM components can result in different mechanical behaviour, including modal response, compared to nominally identical subtractively manufactured components. Past investigations on AM components have shown that modal frequencies are dependent on their build parameters. In a study on a 316 L cantilever beam produced by selective laser melting, Adkins et al. found differences in modal frequencies depending on the build orientation [5]. Similarly, in a different study, the natural frequencies of cantilever beams produced via fused deposition modelling (FDM) from a polylactide polymer were also found to vary with build orientation [6]. The authors attributed the change in resonant frequencies to a change in density with build orientation which led to altered damping characteristics [6]. Nguyen et al. found that modal frequencies increased with improved adhesion between layers in parts manufactured by fused deposition modelling (FDM), a consequence of increased strength resulting from better adhesion [7]. The results of these studies show that AM components exhibit different modal responses depending on build orientation and parameters. As a result, modal analysis has also been proposed as a method for defect identification in 304 L specimens manufactured by selective laser melting [8]. In traditionally manufactured specimens, modal analysis has been used for crack detection in specimens [9].

Residual stresses can affect the dynamic properties of structures by altering their flexural stiffness. In a study on steel plates by Lieven et al. the effect of internal stresses on modal frequencies was evaluated [10]. It was found that the modal frequencies of the plates increased as a result of annealing. During annealing the internal stresses within the plate were reduced, leading to deformation of the formerly flat plate to a slightly curved shape. While annealing did not alter the elastic modulus of the material, the curvature of the plates resulted in an increase in stiffness and led to increased modal frequencies [10]. Similarly, Almeida et al. found that the modal frequencies of reinforced composite plates were drastically altered by introducing residual thermal stresses [11]. Hence the presence of residual stresses and their redistribution during heating, can have a substantial effect on the modal frequencies of a component.

Residual stresses are introduced during both additive and subtractive manufacturing. Large internal stresses are produced during the additive manufacture of metals through the temperature gradient mechanism (TGM) [12]. In L-PDF, components are built layer-by-layer through the selective melting of metal powder with a high-power laser. As the laser rasters over a layer, a steep temperature gradient is formed between the newly molten material and the solid material below. While the high temperature material will expand, its deformation is constrained by the cooler material below it. Hence, a compressive stress develops in the new layer and tensile stresses form in the cooler layers. Conversely during the cool down phase, the newly solidified material will contract, leading to tensile stresses and compressive stresses in the layer below it [13]. Research has shown that, generally, at the end of a build the top and bottom of a built component are in tension while the middle is in compression, resulting in concave deformation relative to the laser beam [14]. Initially the residual stresses result in elastic deformation, however, stresses can accumulate to cause plastic deformation and lead to failure of the component. As a result, support structures and buttresses often need to be incorporated into the build process of a component and are removed post manufacture [15].

The literature reviewed above shows the importance of understanding the modal behaviour of parts produced using additive manufacturing. In this study, geometrically-reinforced thin plates manufactured in Inconel 625 via L-PBF were subject to modal testing before and after heating. Heating caused a change in their shape, and therefore their modal responses. The plates were heated to a nominal temperature of 820 °C for a period of six minutes. Previous investigations have found that annealing of Inconel 625 below 900 °C resulted in negligible changes in hardness and yield stress and some carbide precipitation [2, 16]. Due to the short amount of time the specimens were held at 820 °C, microstructural changes were deemed to be negligible. However, this was sufficient time for the internal stress state of the plates to change causing deformation of the plate and the observed changes in shape. Two specimens were produced via L-PBF in landscape and portrait orientation, while one specimen was subtractively manufactured through machining. The change in shape of the specimens due to heating was evaluated and the effect of this change on modal parameters was measured. It was found that heating the plates produced significant out-of-plane changes in shape, probably as a result of changes in the residual stresses. It was also found that the shape changes caused a shift in the natural frequencies. The behaviour of the plate additively manufactured in the landscape orientation was similar to the plate manufactured subtractively and different to the plate manufactured additively in the portrait orientation. These results imply that careful design of the additive manufacturing process can yield thin parts that perform in a similar manner to their conventionally manufactured counterparts.

## Methods

### Additively Manufactured Specimens

**Fig. 1:**
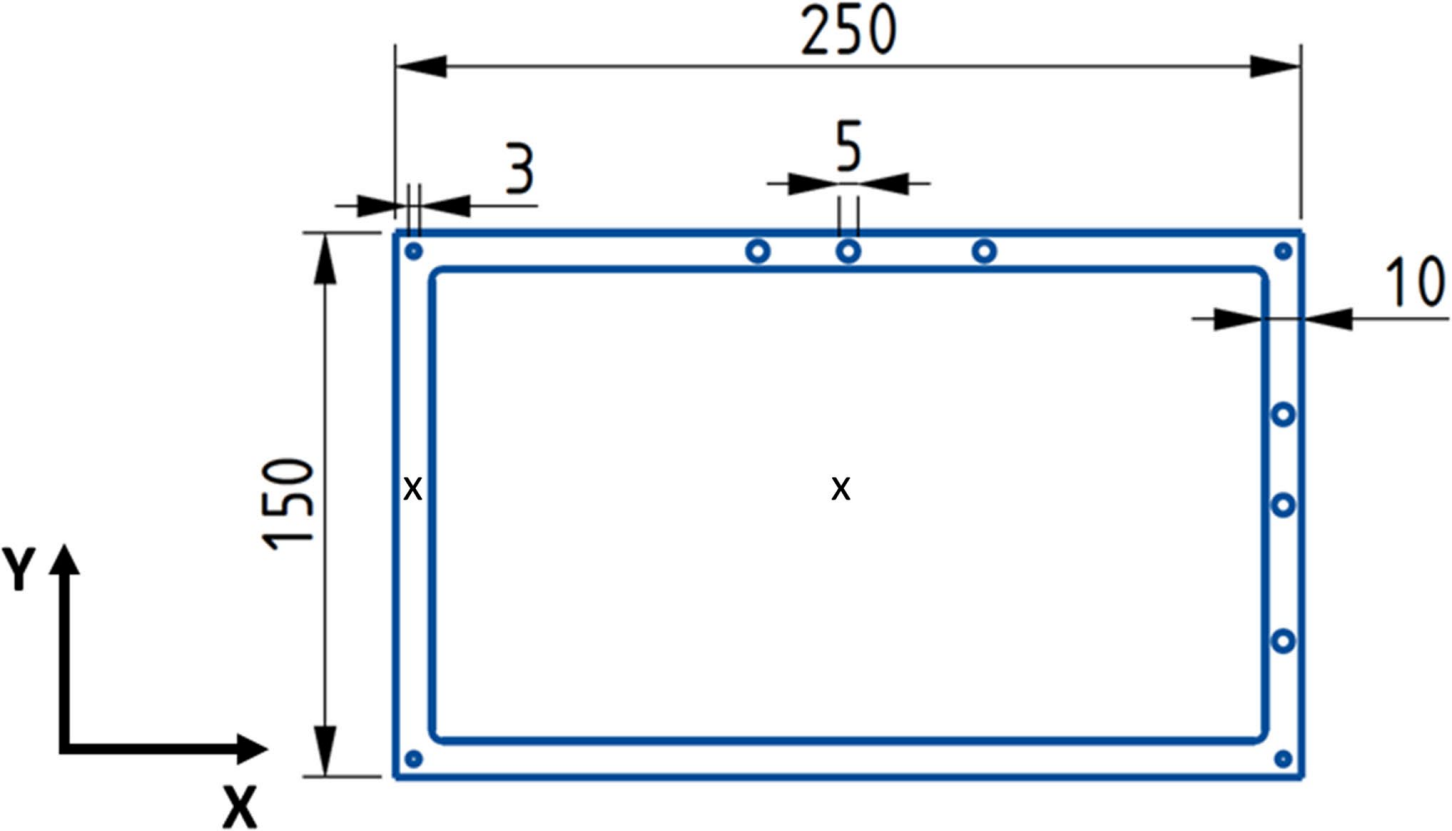
Dimensions of the geometrically-reinforced Inconel 625 plates. All values are given in mm. The marked ‘×’ on the frame and plate interior denote points used to monitor local Z-displacement and temperature during thermal experiments in Figures 5, 6, 7. Full field displacement and temperature were monitored using DIC and thermal cameras. The frame thickness was 4.8 mm while the central plate thickness was 1.2 mm. In the AM plates the build direction was x for portrait and y for landscape

Specimens of the dimensions given in Fig. 1 were additively manufactured via L-PBF with a Renishaw AM250 machine (Renishaw, UK). As seen in Fig. 1, the reinforced plate consisted of a central rectangular area with dimensions of 230 × 130 × 1.2 mm which was supported by a 10 mm wide frame with a thickness of 4.8 mm. Several holes were introduced into the frame to allow different mounting configurations in the modal experiments, however only the central 5 mm hole was used in the experiments described in this study.

The geometrically-reinforced plates were produced in portrait (x-axis build direction) and landscape (y axis build direction) orientations using Inconel 625 gas-atomised powder (Carpenter Additive, UK). To prevent severe outof-plane deformations and build failures, the edges of the specimens were reinforced with buttresses which were later removed. Builds in landscape orientation only required out-of-plane buttresses, while those in portrait orientation required in-plane and out-of-plane buttresses [15]. The specimens were separated from their build plate and supports prior to testing.

The key build parameters are summarised in Table 1 and a detailed description of the manufacturing process can be found in [15]. Previous measurements of relative density produced by this building strategy found a mean relative density of 99.64% with a standard deviation of 0.42% [15]. The relative densities were measured using the Archimedes method from cubes with a side length of 10 mm.

**Table 1:** L-PBF build parameters used to produce geometrically-reinforced Inconel 625 plates with a Renishaw AM 250, from (15)

Scan Strategy	Stripes
Laser Power	400 W
Exposure time	40 µs
Point distance	70 μm
Layer thickness	60 μm
Base plate temperature	170 °C

### Subtractively Manufactured Specimen

A geometrically-reinforced plate was subtractively manufactured to compare its modal and thermal behaviour to that of the AM specimens. The specimen was machined from a 150 × 250 × 4.8 mm Inconel 625 plate (Dynamic Metals, UK) to the dimensions seen in Fig. 1. The edges of the plate were clamped during machining to prevent deformation and warping, following the procedure described by Silva et al. [17].

### Thermal Loading

**Fig. 2:**
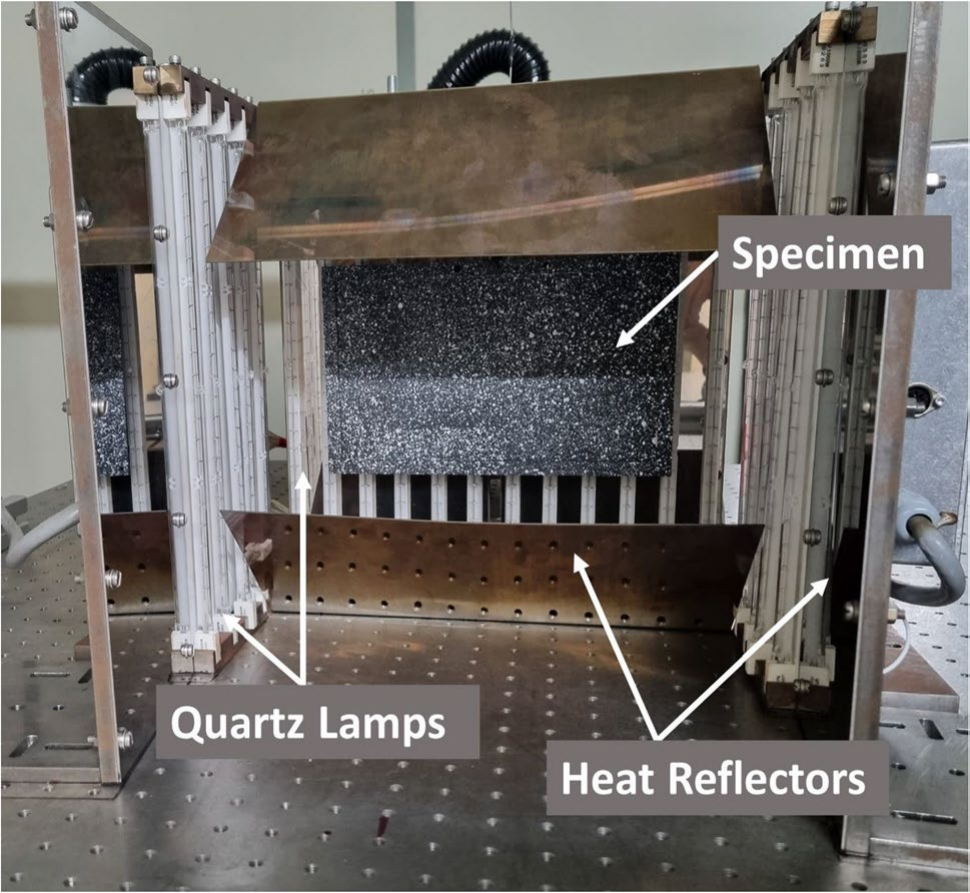
Image of the heating setup used to achieve a nominal temperature of 820 C. The reinforced plate, heat reflectors, and quartz lamps are labelled

Specimens were heated to a nominal temperature of 820 °C using arrays of 1 kW halogen quartz lamps (QIR 240 1000 V2D, Ushio, Steinhöring, Germany). An annotated image of the setup used to heat the specimens is shown in Fig. 2. An array of 12 quartz lamps was placed behind the specimen with a nominal distance of 1 mm between the reinforced plate and the heat lamps. Another two arrays containing five quartz lamps each were placed to the sides of the specimen. Additionally, heat reflectors were placed underneath, above, and to the sides of the specimen. This setup was intended to increase the amount of infrared (IR) radiation directed at the specimen relative to the arrangement employed by Silva et al. [17].

The quartz lamps were set to their maximum power and the evolution of temperature with time across the specimen was monitored with a micro-bolometer (TIM 400, MICRO-EPSILON UK, Birkenhead, UK). The micro-bolometer was located 210 cm from the specimen, leading to a resolution of 0.8 px/mm. Due to the calibration options of the thermal camera, only specific temperature ranges could be monitored. For the temperatures achieved in this work, the most suitable temperature range was 150 °C to 900 °C. Temperatures over 150 °C were of greatest interest in this investigation and only a few seconds of heating data were lost until the specimen reached 150 °C. The reinforced plate was heated for nominally 6 min, with thermal equilibrium being achieved within 4 min of heating. At thermal equilibrium, a relatively uniform temperature was observed across all specimens (Figure S1). Thermal images and digital image correlation (DIC) data were collected for a total of half an hour, so that both the heating and cooling of the plate were monitored.

### Modal Tests

**Fig. 3:**
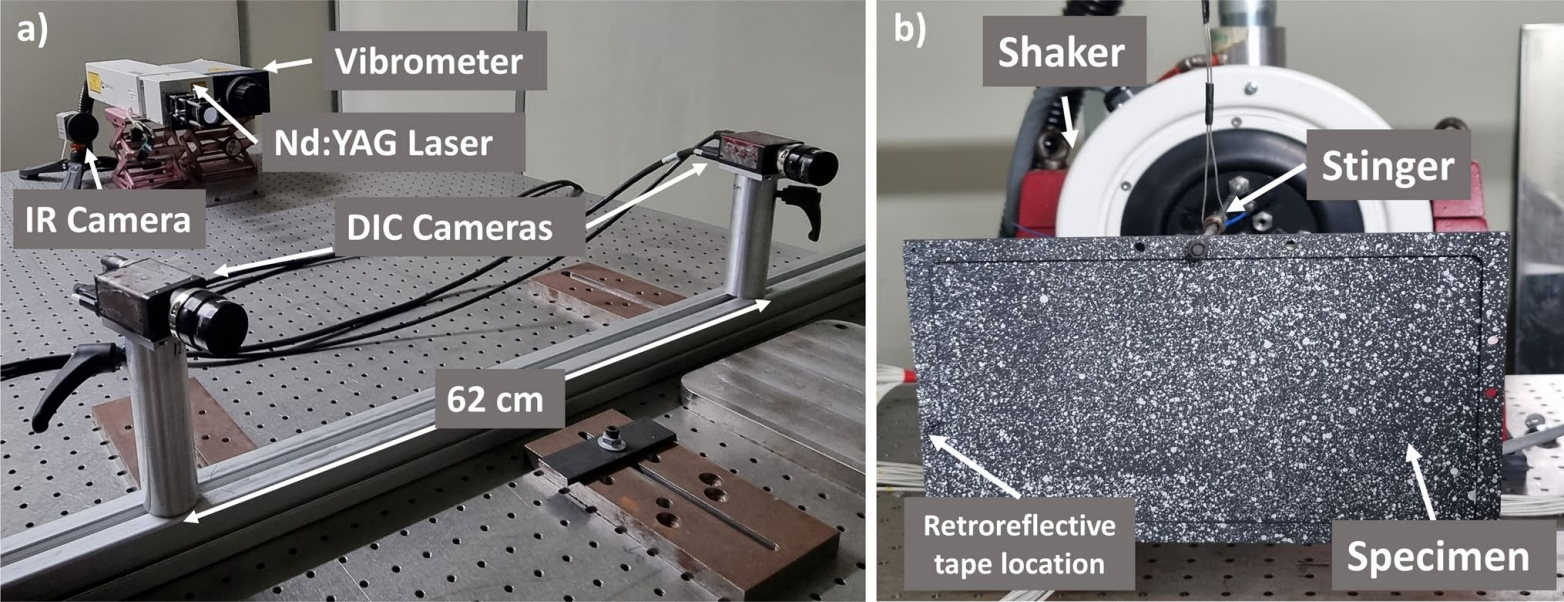
Setup of the system used for modal tests and to determine the resonance frequencies of the specimen. **a** shows the view from adjacent to the specimen towards the DIC cameras, IR camera, Nd:YAG Laser and vibrometer. In (**b**) the view from the measurement system towards the specimen that is connected to the shaker via a stinger is shown

Following the heating and cooling cycle, modal tests were conducted to find the resonant frequencies and modal shapes of the geometrically-reinforced plates. The setup employed in the modal tests is shown in Fig. 3. The DIC system, laser, vibrometer and IR camera can be seen in Fig. 3a. The modal testing system consisted of the vibrometer (OFV-503, Polytec GmbH, Waldbronn, Germany), a 1 kW amplifier (DSA1-1 K, DataPhysics), and a shaker (V100, DataPhysics, San Jose, CA, USA). As seen in Fig. 3b, the specimen was connected to the shaker via a metal stinger rod threaded into the shaker and bolted at the middle top hole in the frame. To determine the resonant frequencies, a broadband signal between 0 and 1000 Hz was applied to the specimen through a vibration controller (ABACUS, DataPhysics). An accelerometer was attached to the shaker to measure the input signal of the system. The output signal was captured via the vibrometer, which was directed at a piece of retroreflective tape attached to the frame of the plate. A signal analyser (SignalCalc, Data Physics) then processed the input and output signals to compute a transfer function. To identify the resonant frequencies of the specimen, the peaks of the transfer function were found. An example of the transfer function showing the resonance frequencies of an AM specimen manufactured in landscape can be found in the supplementary data (Figure S2).

To determine modal shapes, the specimens were then harmonically loaded at their resonant frequencies, obtained from the frequency response function, with a sinusoidal wave produced by a function generator. Pulsed laser digital image correlation (PL-DIC) [18] was then performed to capture the modal shapes. It was found that at lower frequencies the modal shapes were more pronounced than at higher frequencies, primarily because of degrading of the signal-to-noise ratio with increasing frequency. To reduce noise in the observed modal shapes, the signal amplitude for the shaker was increased incrementally with frequency.

### Pulsed Laser Digital Image Correlation (PL-DIC)

Full-field displacement data were collected via a PL-DIC system in both the thermal loading and modal experiments. The PL-DIC setup included a pulsed Nd: YAG laser (Nano L-200, Litron, Rugby, UK) as a light source and DIC cameras, as shown in Fig. 3a. To achieve good illumination of the test area, lenses and diffusers were placed in front of the laser beam. The laser produced a 4 ns pulse of green light at a wavelength of 532 nm. Data were collected with a commercially-available DIC system (Q-400 system, Dantec Dynamics GmbH, Ulm, Germany). The DIC system cameras (2 MP Stingray F-201b, Allied Vision Technologies GmbH, Stradtroda, Germany) were located 105 cm from the specimen, leading to a resolution of 6.64 px/mm. Additionally, the cameras were fitted with optical bandpass filters with a centre wavelength of 532 nm and a bandwidth of 4 nm to ensure that only light matching the wavelength of the laser reached the sensors of the cameras. As a result of the filter, the cameras were not saturated by the quartz lamps or the glow of black body radiation produced during heating. A basecoat of high temperature black paint (VHT Black, Autotek, James Briggs Ltd, Oldham, UK) was applied to both sides of the specimen for improved absorption of incident IR energy from the heat lamps. Speckle patterns for DIC were produced with white high temperature paint (VHT Flameproof, Cleveland, Ohio, USA), see pattern in Fig. 3b.

To evaluate changes in the shape of the geometrically-reinforced plates caused by heating, static DIC images were captured prior to and after heating without mechanical loading. During thermal loading, DIC data was also collected for half an hour to monitor specimen heating and cooling. Similarly, changes in modal frequencies and shapes were evaluated prior to and after heating. The sinusoidal wave produced by the function generator during harmonic loading was also routed to a timing box, which triggered the pulsed laser and image acquisition simultaneously. A total of 100 images were captured at 9-degree intervals, corresponding to two and a half wave cycles. This allowed modal shapes to be found corresponding to the maximum deflection of the plate.

## Results

### Deformation as a Result of Thermal Loading

**Fig. 4:**
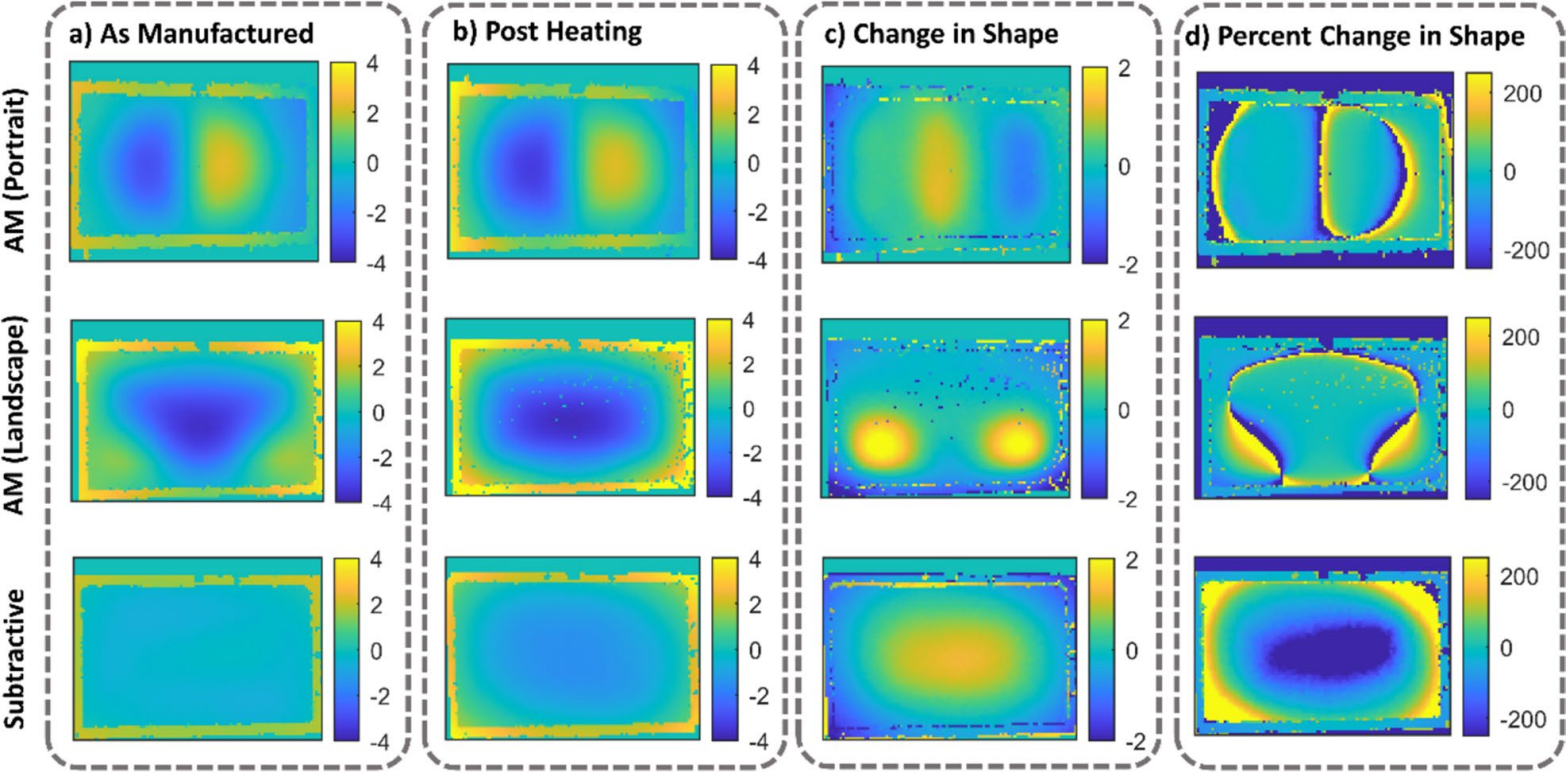
Distance from a reference plane perpendicular to the z-axis of additively and subtractively manufactured geometrically-reinforced plates (**a**) before and (**b**) after heating. In (**c**) the shape change from heating can be seen. The units of the colour bars are given in mm, (**d**) depicts the shape change as a percentage

The shapes of the plates before and after thermal loading were evaluated from the DIC images. Figure 4a shows the distance from a reference plane perpendicular to the z-axis of the AM specimens and the subtractively manufactured specimen in an as-manufactured state. Figure 4b shows the distance from the reference plane of the specimens after thermal loading. Finally, Fig. 4c indicates the change in shape of the plates due to thermal loading. Both Fig. 4a and b show that the shape of the AM specimens varied with their build orientation. In its as manufactured state, the specimen printed in portrait orientation had an “S” shape; with negative and subsequently positive distances in the Z-direction. On the other hand, building in the landscape orientation produced a “U” shape where the material curved into the negative Z-direction only. As seen in the “Change in Shape” column of Fig. 4c, all of the plates underwent significant deformation as a result of heating. The overall shape of the specimens that were manufactured subtractively and additively in the portrait orientation did not change due to heating, but exhibited greater deviation from a flat plane. The AM specimen manufactured in the landscape orientation became symmetrical about both the x- and y-axes as a result of heating. As a result, the shapes of the AM plate built in the landscape orientation and subtractively had a single central dome-shape whereas the plate manufactured in the portrait orientation had a peak and valley shape.

### Displacements during Thermal Loading

**Fig. 5:**
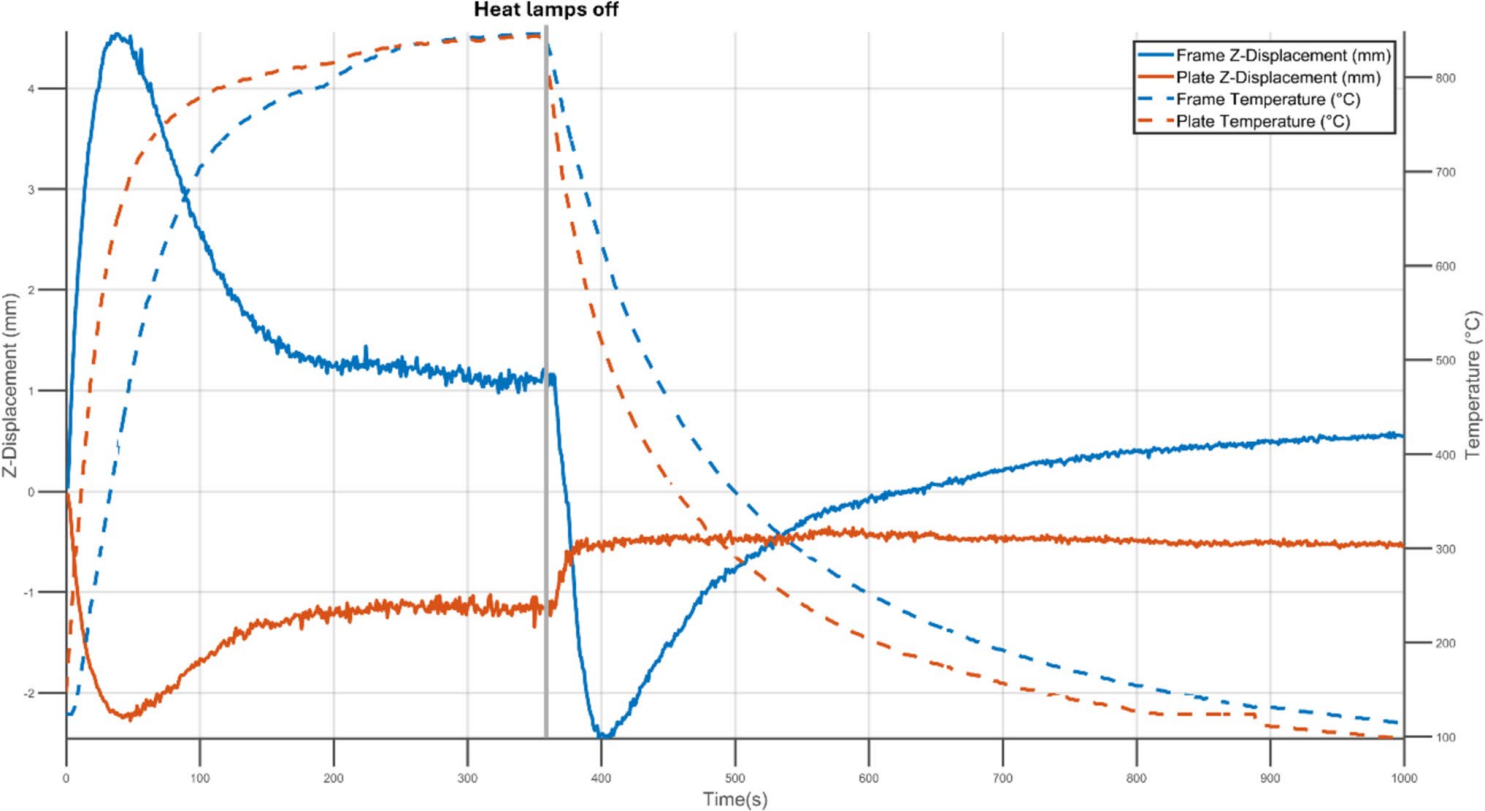
Z-Displacement with rigid body motion removed (solid lines) and temperature (dashed lines) experienced by the frame (blue) and central portion of the plate (orange) of a subtractively manufactured specimen during heating and cooling

To obtain a better understanding of the thermal response of the specimens, DIC data were collected during the heating process. Figure 5 shows the time history of temperature and Z- displacement with rigid body motion removed (RBMR) for the subtractively manufactured plate and frame. The approximate locations used for measurements are marked in Fig. 1. Due to its lower thickness and the consequent lower heat capacity per unit area, the central portion of the specimen experienced a faster temperature increase than the 10 mm-thick reinforcing frame. However, the frame experienced greater deformations than the central plate, with the two parts deforming in opposite directions. After 36 s of heating, both the centre of the plate and surrounding frame reached their peak deflections. At later times, the rate of out-of-plane deflection reduced in both, despite a continuing increase in temperature. This may be associated with the constraint to lateral expansion that the colder, and hence stiffer, frame provided to the central plate. As the temperature difference between the frame and central plate reduced, the effects of the constraint provided by the frame would have lessened and may have led to the observed spring back behaviour [17].

When the heat lamps were turned off after 360 s of heating, the opposite trend was observed. The displacement of the frame changed towards the negative Z-direction, reaching a minimum at 400 s. Beyond 400 s a similar spring back behaviour that was seen during the heating stage could be observed. The central portion of the specimen did not experience this sudden deformation and reached an equilibrium within 40 s of cooling.

**Fig. 6:**
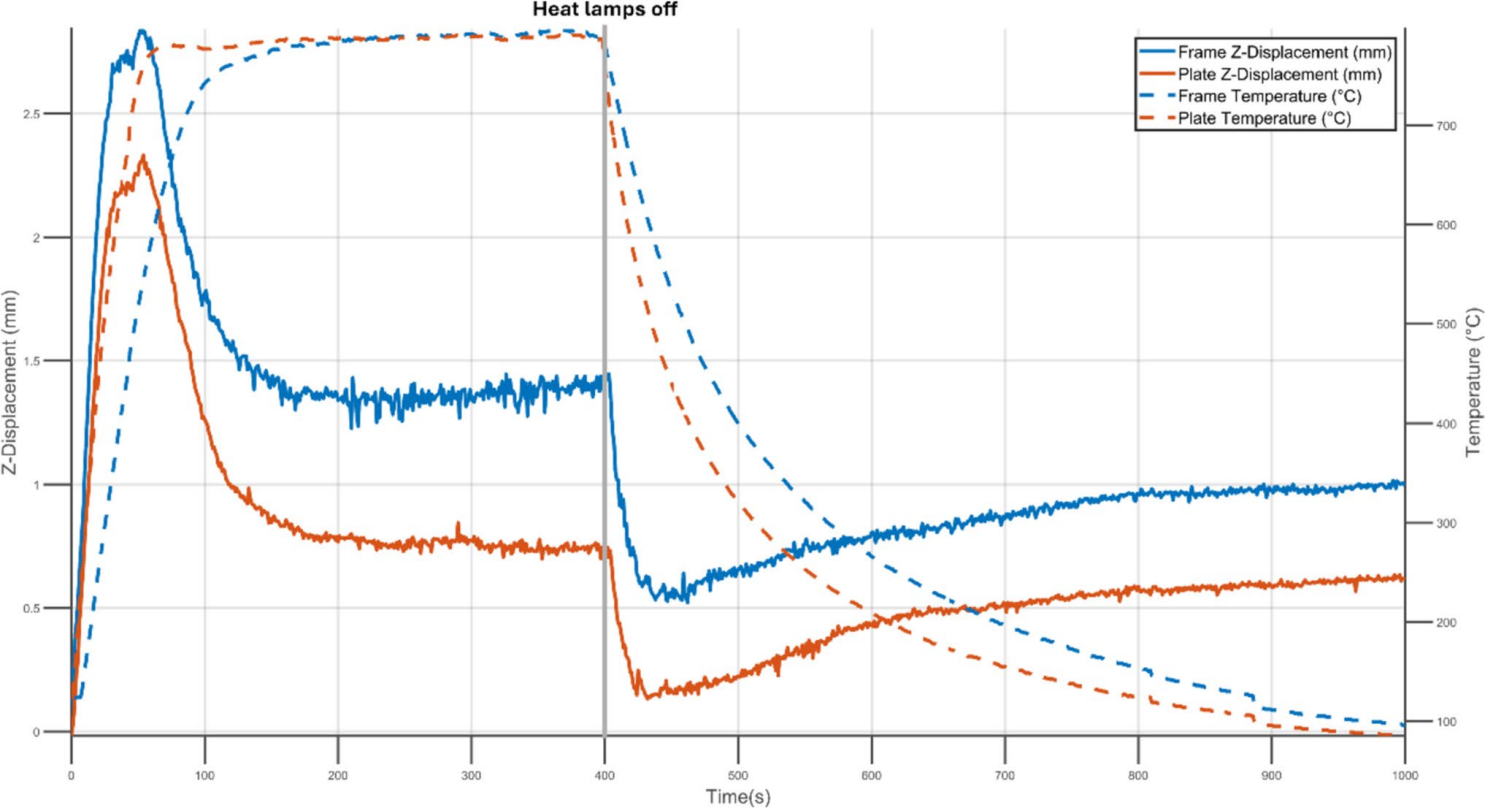
Z-Displacement with rigid body motion removed (solid lines) and temperature (dashed lines) experienced by the frame (blue) and plate (orange) during heating and cooling of an AM specimen manufactured in the portrait orientation

The additively-manufactured specimen built in the portrait orientation exhibited similar behaviour to its subtractively manufactured counterpart as seen in Fig. 6. Both the frame and the central portion of the plate reached a peak displacement after 48 s of heating. Both the plate centre and frame deformed in the same direction. This is an artefact of the original plate shape and the measurement points chosen to describe the displacement of the plate. When the quartz lamps were turned off, the displacements rapidly decreased before reaching a steady state value.

**Fig. 7:**
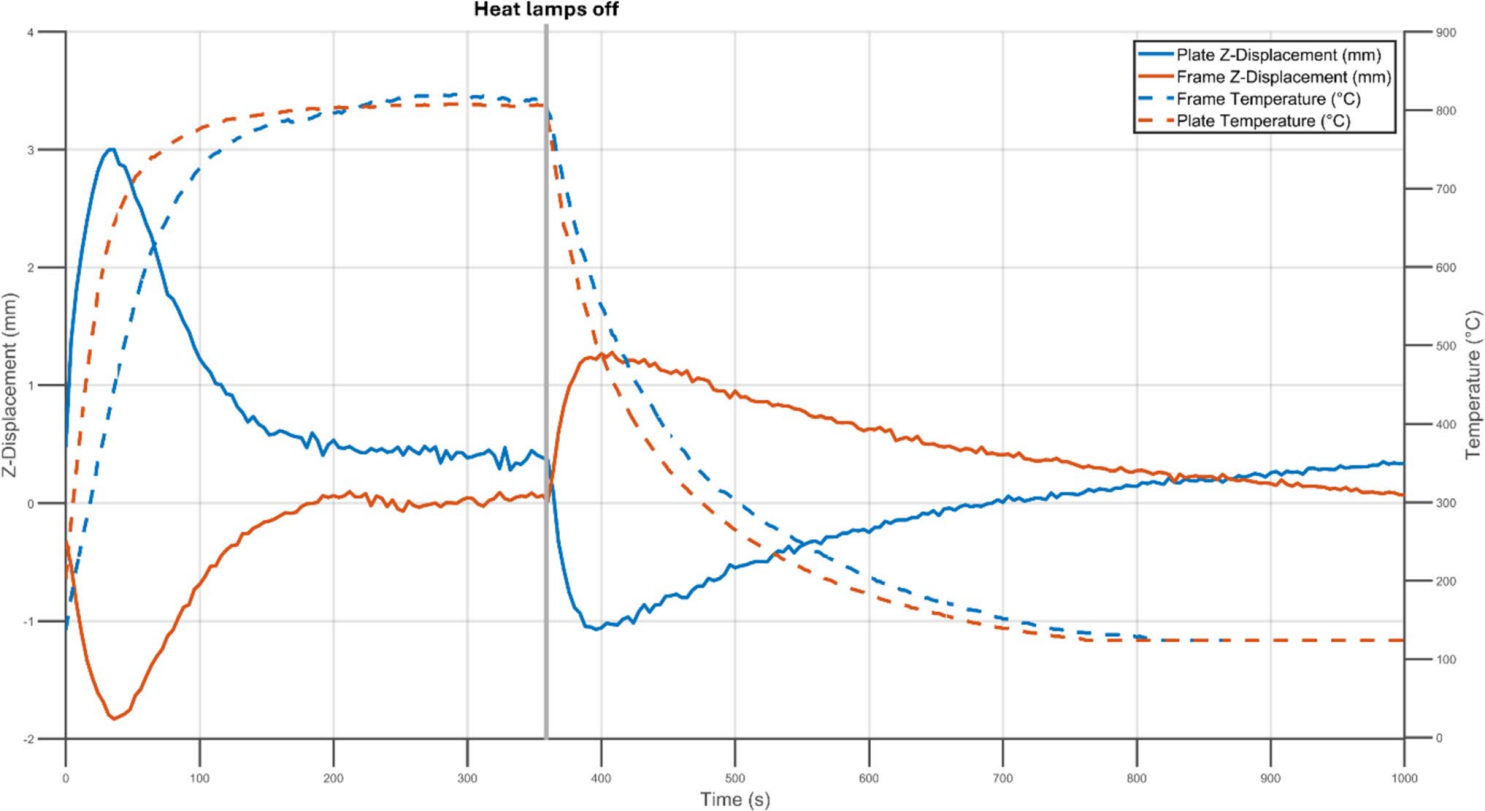
Z-Displacement with rigid body motion removed (solid lines) and temperature (dashed lines) experienced by the frame (blue) and plate (orange) during heating of an AM specimen manufactured in the landscape orientation

The Z-deformation (RBMR) and the temperature of the AM specimen built in the landscape orientation is given in Fig. 7. During the heating stage of the test, the plate displaced in a similar manner to the subtractively manufactured specimen. The maximum displacements were reached after 32 s of heating, after which displacements started to reduce. When the heat lamps were turned off, the displacements moved back a near zero baseline value.

### Harmonic Loading

**Fig. 8:**
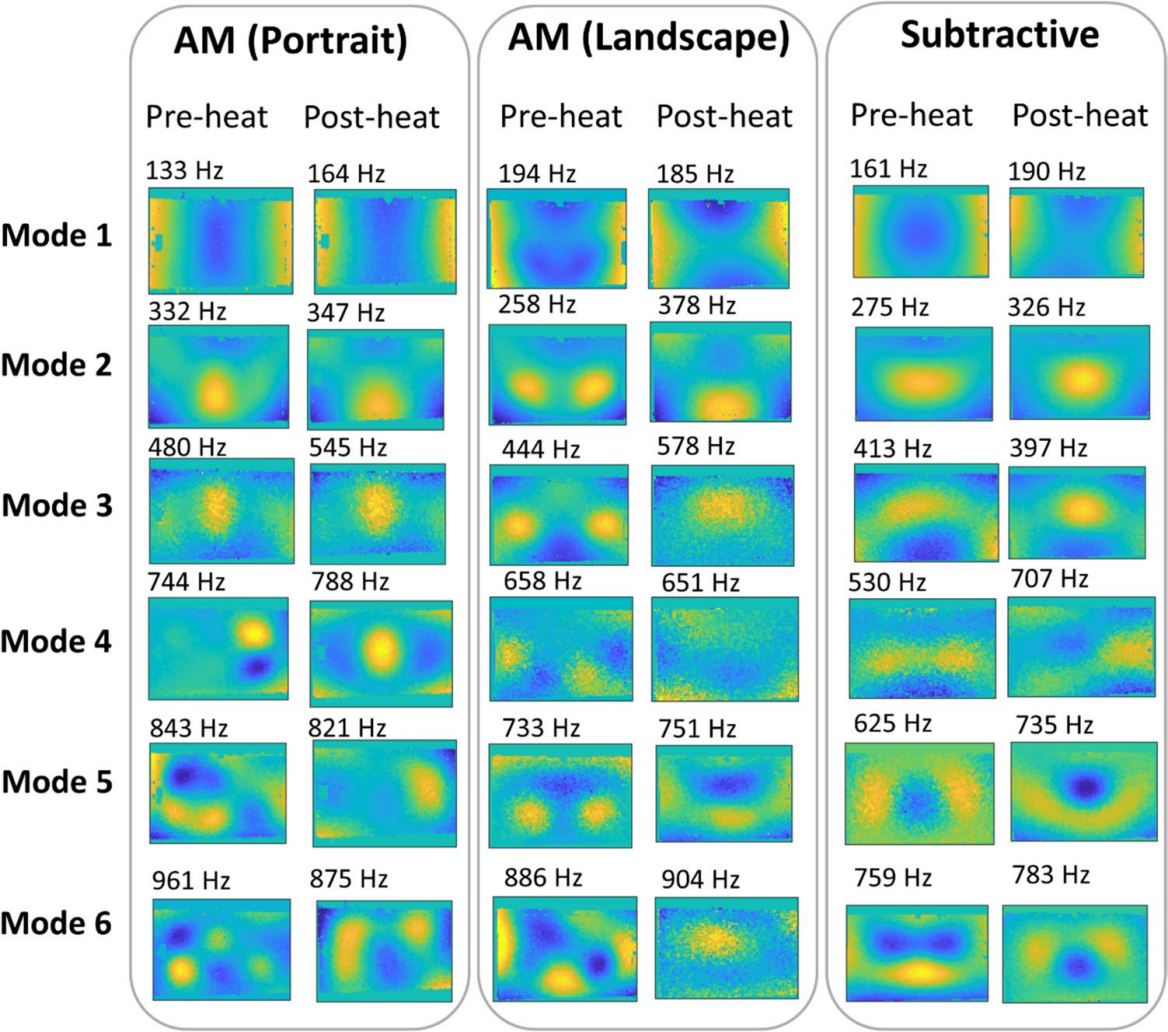
Modal shapes and frequencies of additively and subtractively manufactured specimens before and after heating

 The significant deformations experienced by the geometrically-reinforced specimens were expected to create a shift in all of the modal frequencies and shapes as compared to the preheated state. In Fig. 8 the modal frequencies and shapes measured between 0 and 1000 Hz for the additively and subtractively manufactured specimens before and after heating are given. The modal frequencies were identified from the frequency response function of each specimen, as explained in the "Modal Tests" section and shown in Figure S2, and then each specimen was excited at each of its natural frequencies to obtain the corresponding modal shape using the pulsed-laser digital image correlation system. Repeated thermal loading did not change the specimen shape (see Figure S3) or the modal frequencies. It was found that for the first three resonant frequencies, the modal shapes were similar in all specimens regardless of their thermal history (see Fig. 8) ; however, for higher resonant frequencies, the modal shapes differed substantially between specimens. In particular, differences could be seen between specimens before heating. As a result of thermal cycling, the AM specimen manufactured in the landscape orientation and the subtractively manufactured specimen had near identical mode shapes.

**Fig. 9:**
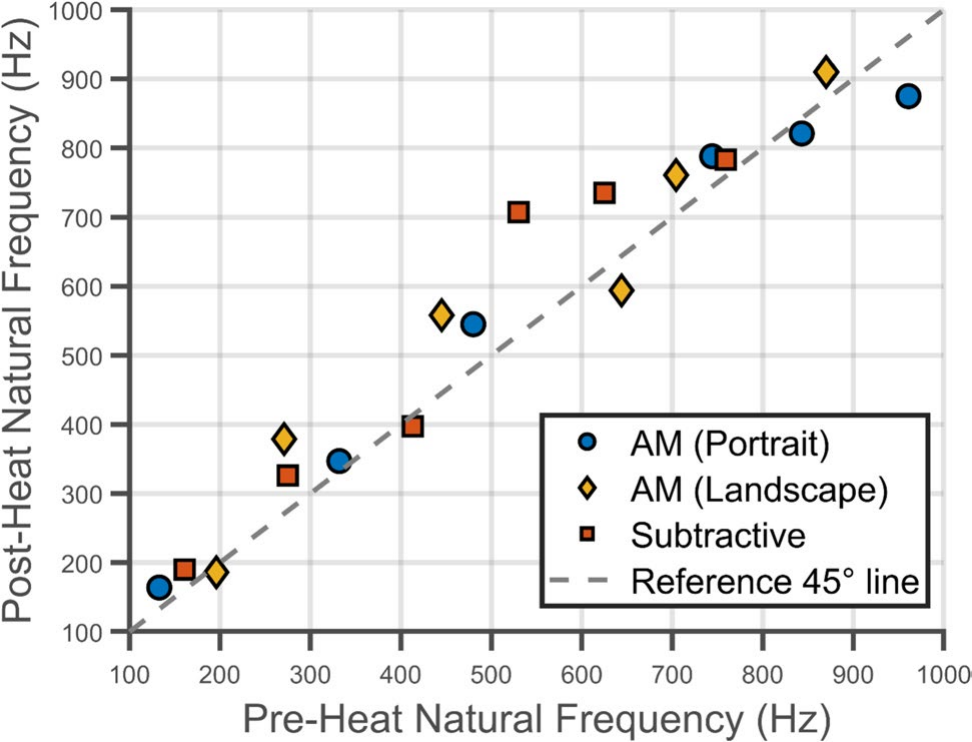
Comparisons of the modal frequencies of AM specimens manufactured in portrait (circles), in landscape (diamonds), and subtractively (squares)

In most cases, thermal loading resulted in an increase in frequency for each resonant mode. Figure 9 shows the resonant frequencies of all of the tested specimens before and after heating. A reference 45° line has been added and represents no change in resonance frequency due to heating. In other words, all data points above the reference line represent an increase in natural frequency as a result of heating. There is little change in natural frequencies for the first two modes; however, for the higher order modes there are significant differences which could be a result of experimental scatter or mode shifting and mode switching [19].

## Discussion

The first thermal cycle after manufacturing led to out-of-plane deformations of up to 4 mm in both the subtractively and additively manufactured plates. The shapes of the AM specimen that was built in the landscape orientation and the subtractively manufactured specimen showed a high degree of similarity after thermal loading. In both cases, there was out-of-plane deflection in opposite directions at the edges compared to the centre. The change in out-of-plane displacement caused by thermal cycling is indicative of a redistribution and reduction of internal stresses. Even though the plates were exposed to temperatures used in the heat treatments of Inconel 625, no recrystallisation or extensive carbide precipitation was anticipated to occur over a six minute period [2, 16]. Hence, only changes in the internal stress states of the material were expected. It was found that performing repeated heat cycling tests did not lead to significant changes in the specimen shapes beyond the first cycle (see Figure S3). This confirmed that thermal loading of the as-manufactured specimens led to a change in the residual stress state.

During thermal loading, the temperatures across the specimens were recorded with an IR camera and displacements in the Z-direction were measured using DIC. Across all specimens the central portion of the plate heated and cooled at faster rates then the frame. This was expected as the frame was three times as thick as the plate and therefore had a larger heat capacity which required more energy transfer to reach the same temperature. Nevertheless, both reached peak magnitudes of deformation simultaneously during the heating stage. By surrounding the central portion of the specimen and heating at a slower rate, the frame acted as a mechanical constraint to the interior displacement of the central plate. Conversely, as the frame cooled slower the mechanical constraint it imposed on the central plate reduced. As a result, the frame played a key role in the displacement behaviour of the entire specimen.

**Fig. 10:**
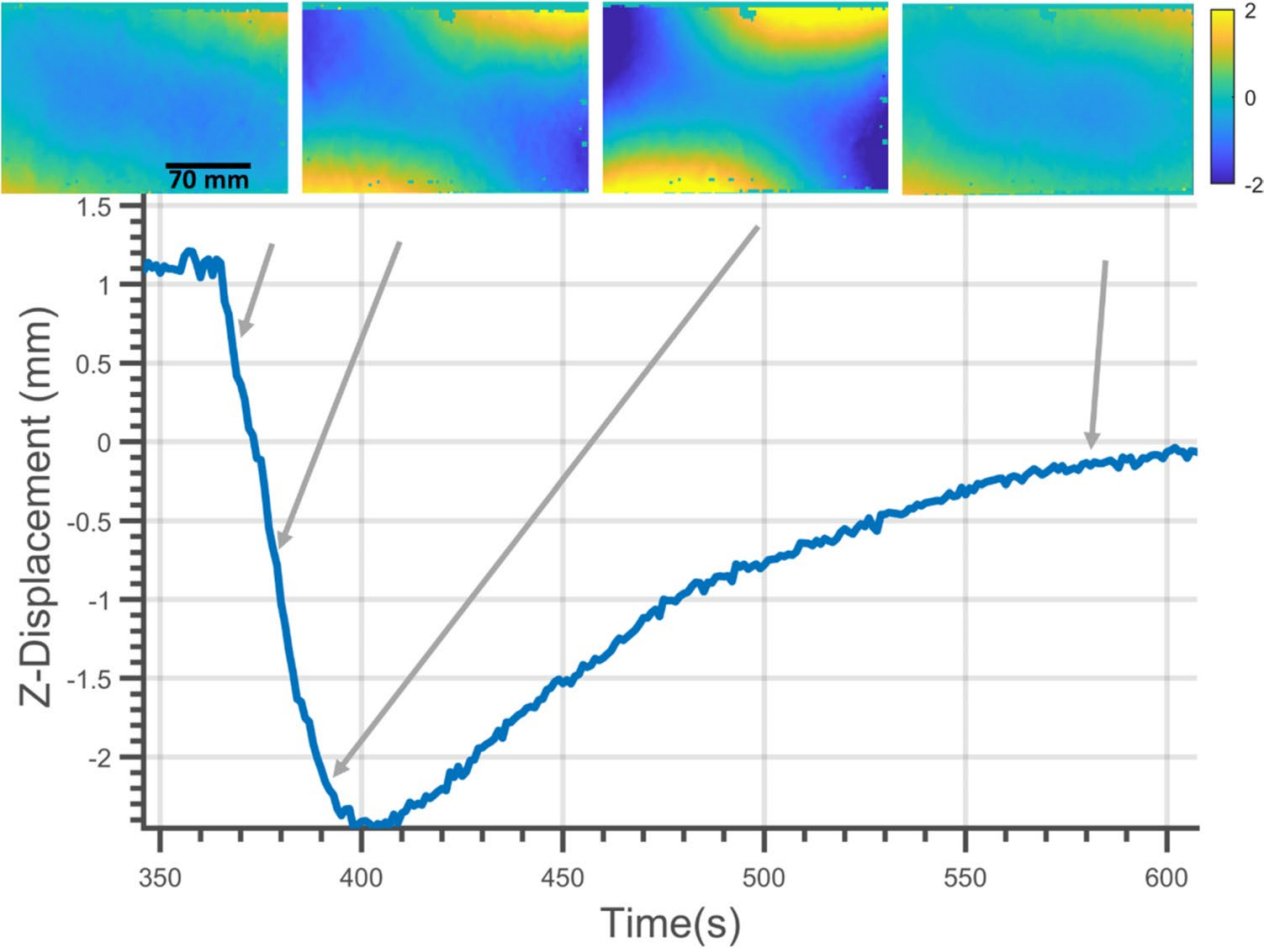
Deformation of the subtractively manufactured plate during the initial stages of cooling. DIC images of Z-Displacement (out-of-plane), with rigid body motion removed, at four different times are shown. The scale on the colour bar shows Z-Displacement (RBMR) in mm

In the first 40 s of cooling, the subtractively manufactured specimen underwent significant but temporary warping. In Fig. 5 this is evident as a reduction in the frame displacement that occurred between 360 and 400 s. The change in the deformation of the subtractively manufactured specimen during the cooling stage can be seen in Fig. 10. Within the first 15 s of cooling, the edges of the frame began warping. The warping magnitude of the displacement increased progressively up to 400 s. Beyond 400 s, the amount of deformation reduced until a steady-state was reached. This behaviour may be attributed to the manufacturing method used in the production of the specimen. The subtractively manufactured plate was made by progressively removing material with a cutting tool while clamping the frame at eight points around its perimeter (four corners and the mid-point of each edge), which may have induced large gradients of residual stress at the interface between the frame and the plate. The removal of material in the subtractive manufacturing process was inevitably asymmetric as consequence of the constraints on machining with a single cutting tool and thus, it is likely that the residual stresses induced would be asymmetric and lead to the asymmetric deformation seen Fig. 10.

**Fig. 11:**
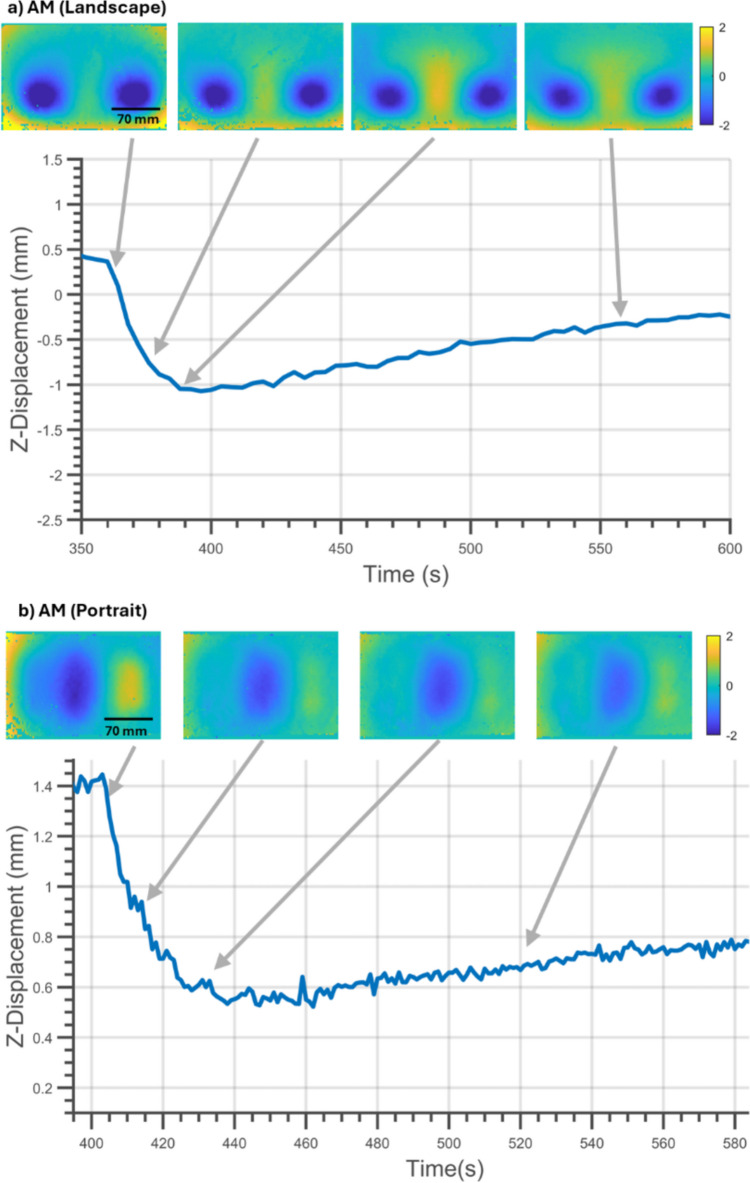
Deformation of the AM plate manufactured in (**a**) landscape and (**b**) portrait orientation during the initial stages of cooling. DIC images of Z-Displacement (RBMR) at four different times are shown. The scale on the colour bar shows Z-Displacement (RBMR) in mm

The phenomenon seen in Fig. 10 was not observed in the two additively manufactured specimens. Figure 11 shows the out-of-plane displacements in the initial stages of cooling of the additively manufactured specimens. In Fig. 11a, a monotonic decrease in the displacement with cooling can be seen from 360 to 400 s in the specimen manufactured in the landscape orientation. The displacement then increased until an equilibrium state was reached. A similar decrease in displacement was observed in the specimen manufactured in the portrait orientation as seen in Fig. 11b.

Modal analysis of all of the reinforced plates showed that the modal shapes corresponding to the first three resonant frequencies were the same. For most modes, the resonant frequencies shifted upwards after heating. This suggests an increase in stiffness of the specimen as a result of the change in shape due to the stress redistribution. These results are similar to those found by Lieven et al. where resonant frequencies increased as a result of annealing [10]. Additionally, the modal shapes of the additively manufactured specimen built in the landscape orientation and the subtractively manufactured specimen resembled each other after heating. This may be the result of a change in the internal stress state of the specimens after thermal loading, which led to both specimens to become similar in shape (see Fig. 4). Hence, results suggest that heat treatments of additively manufactured materials can lead components to more closely follow the dynamic behaviour of their subtractively manufactured counter parts. These data demonstrate the significance of internal stress states on modal behaviour. Additively manufactured components could therefore be designed and manufactured for more favourable dynamic behaviour by selectively altering their residual stresses.

## Conclusions

The effect of heating on the subsequent modal response of geometrically-reinforced thin plates produced via laser powder bed fusion (L-PBF) of Inconel-625 was investigated for the first time in a series of experiments. Additively manufactured specimens were produced in landscape and portrait orientations and results were compared to a subtractively manufactured specimen. The reinforced plates were heated to a nominal temperature of 820 °C and the evolution of temperature with time across the specimen was monitored with an IR camera. Displacements and modal shapes were evaluated pre- and post- heating using pulsed-laser digital image correlation (PL-DIC). These data will provide important information for the validation of future simulation work.

The results showed that applying a heating cycle up to nominal temperatures of 820 °C resulted in significant out-of-plane shape changes of the order of 4 mm in the specimens. The observed change in shape of the 150 × 250 mm specimens due to heating is indicative of changes in residual stresses induced during the manufacturing process. The IR data collected during heating showed a uniform temperature distribution across the plates. It was found that the thin plate consistently experienced a larger rate of temperature increase and decrease than the thick surrounding frame. Nevertheless, both plate and frame experienced their maximum deformations at the same time and within the first minute of heating. Conversely, when the heat source was removed the plate cooled down faster than the frame, with the frame acting like a heat sink. It is likely that the thick frame mechanically constrained the deformation of the thin plate during the heating and cooling process. This mechanism is similar to those proposed previously for reinforced-plates of similar geometry made using conventional machining [17].

The modal response of the geometrically-reinforced plates was measured before and after heating. It was observed that there was a weak trend for the heating to increase the natural frequencies probably as a result of shape changes causing stiffening of the plates. The manufacturing process and builtdorientation had little effect on natural frequencies for the first two modes; however, it appeared to have a greater effect for higher modes. Additionally, the plate manufactured in landscape orientation had approximately the same modal shapes as the subtractively manufactured specimen after heat cycling, which was likely due to the similarity in their out-of-plane displacements. This suggests that it is possible to design the additive manufacturing process to yield parts with a similar performance to their subtractively manufactured counterparts.

Ultimately, the natural frequencies and modal shapes are greatly influenced by the residual shape which has been found to be a function of the manufacturing process and the built orientation which in turn will affect residual stresses. Hence, it can be concluded that there is potential for designing AM parts with residual stresses to produce a desired dynamic response.

## Supplementary Information

Below is the link to the electronic supplementary material.

High resolution image(TIF 9410 KB)

(TIF 2129 KB)

High Resolution image(TIFF 5030 KB)

## Data Availability

All data generated in this study can be found with the following 10.17632/mpgzfwr7fk.1.
