# Supplementary figures and images for: A Comparative Study of the Modal Response of Additively and Subtractively Manufactured Thin Plates After Thermal Loading
Source: Exp Mech. 2025 Jan 23;65(4):481–93. doi: 10.1007/s11340-024-01130-5 (PMC12058964; doi:10.1007/s11340-024-01130-5)

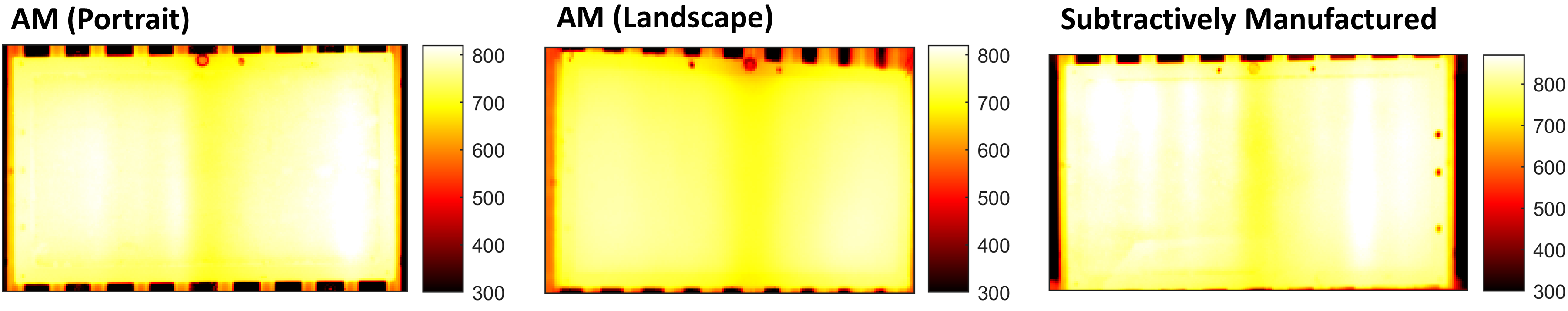

Supplement: Supplementary file 1 — High resolution image(TIF 9410 KB) [file 11340_2024_1130_MOESM1_ESM.tif]

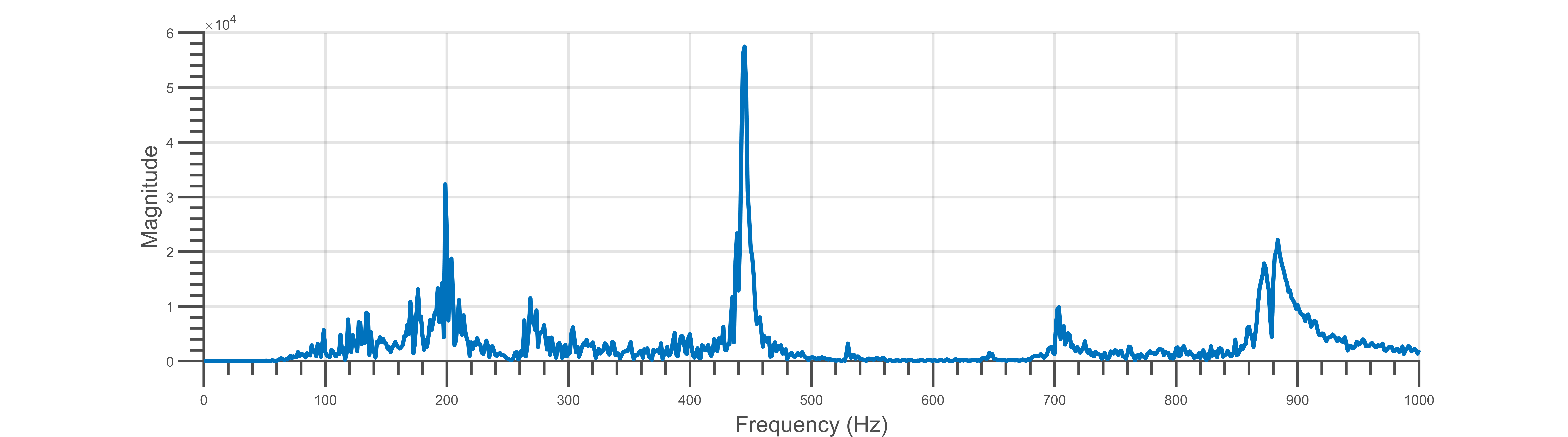

Supplement: Supplementary file 2 — (TIF 2129 KB) [file 11340_2024_1130_MOESM2_ESM.tif]

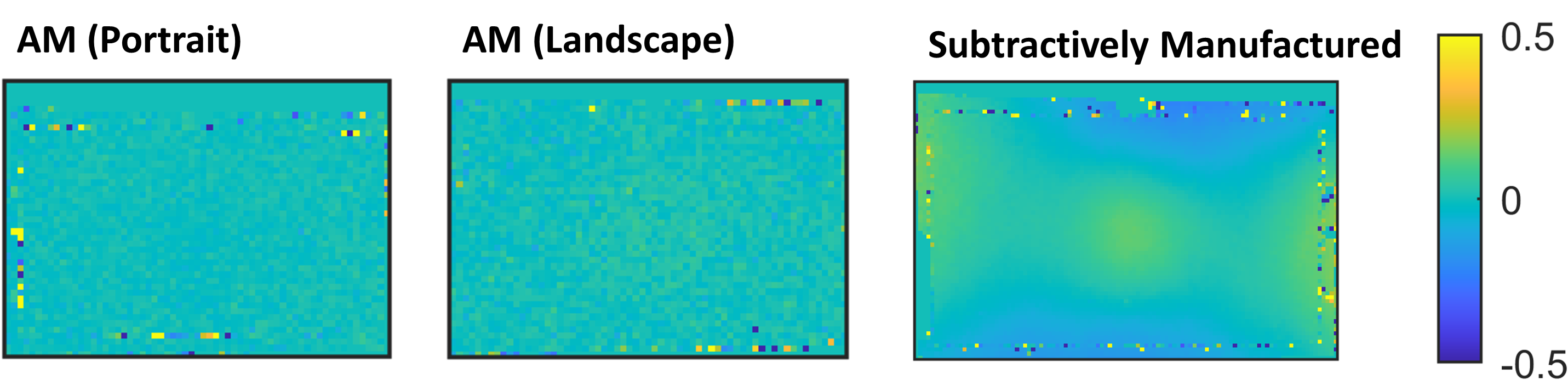

Supplement: Supplementary file 3 — High Resolution image(TIFF 5030 KB) [file 11340_2024_1130_MOESM3_ESM.tif]
